# Three-Dimensional Volume Rendering of Pelvic Floor Anatomy with Focus on Fibroids in Relation to the Lower Urogenital Tract Based on Cross-Sectional MRI Images

**DOI:** 10.1007/s10916-023-01947-y

**Published:** 2023-05-12

**Authors:** Ka Siu Fan, Constantin Durnea, Christiana Campani Nygaard, Miriam Khalil, Stergios K. Doumouchtsis

**Affiliations:** 1 Institute for Medical and Biomedical Education, St. George’s University of London, London, UK; 2 Department of Obstetrics and Gynaecology, Epsom & St Helier University Hospitals NHS Trust, St George’s University of London, London, UK; 3 Luton and Dunstable University Hospital, Bedfordshire Hospitals NHS Foundation Trust, Luton, UK; 4 Department of Obstetrics and Gynecology, Hospital Sao Lucas, Pontifícia Universidade Católica Do Rio Grande Do Sul, Porto Alegre, Brazil; 5 Department of Radiology, Epsom & St Helier University Hospitals NHS Trust, London, UK; 6 Laboratory of Experimental Surgery and Surgical Research N. S. Christeas, National and Kapodistrian University of Athens, Medical School, Athens, Greece; 7 School of Medicine, American University of the Caribbean, Pembroke Pines, FL USA; 8 School of Medicine, Ross University, Miramar, FL USA

**Keywords:** Magnetic resonance imaging (MRI), Uterine fibroids, Lower urinary tract symptoms (LUTS), 3D pelvic model, 3D volume rendering, Image processing, 3D Slicer, OsiriX

## Abstract

We aimed to assess the feasibility of developing three-dimensional (3D) models of pelvic organs using cross-sectional MRI images of patients with uterine fibroids and urinary symptoms and of obtaining anatomical information unavailable in 2D imaging modalities. We also aimed to compare two image processing applications. We performed a feasibility study analysing MRI scans from three women, aged 30 to 58 years old, with fibroids and urinary symptoms. Cross-sectional images were used to render 3D models of pelvic anatomy, including bladder, uterus and fibroids, using 3D Slicer and OsiriX. Dimensions, volumes and anatomical relationships of the pelvic organs were evaluated. Comparisons between anatomical landmarks and measurements obtained from the two image processing applications were undertaken. Rendered 3D pelvic models yielded detailed anatomical information and data on spatial relationships that were unobtainable from cross-sectional images. Models were rendered in sufficient resolution to aid understanding of spatial relationships between urinary bladder, uterus and fibroid(s). Measurements of fibroid volumes ranged from 5,336 to 418,012 mm^3^ and distances between the fibroid and urinary bladder ranged from 0.10 to 83.60 mm. Statistical analysis of measurements showed no significant differences in measurements between the two image processing applications. To date, limited data exist on the use of 3D volume reconstructions of routine MRI scans, to investigate pelvic pathologies such as fibroids in women with urinary symptoms. This study suggests that post-MRI image processing can provide additional information over standard MRI. Further studies are required to assess the role of these data in clinical practice, surgical planning and training. Three-dimensional reconstruction of routine two-dimensional magnetic resonance imaging provides additional anatomical information and may improve our understanding of anatomical relationships, their role in clinical presentations and possibly guide clinical and surgical management.

## Introduction

Uterine fibroids are benign tumours that demonstrate varying grades of smooth muscle differentiation. Fibroid is the most common pelvic tumour among young women, with a prevalence of 5%-21% and incidence of 20%-40% [1]. Fibroids can cause lower urinary tract symptoms (LUTS) such as urinary incontinence and voiding dysfunction [2]. LUTS can affect women’s quality of life, with up to 60% of women with fibroids experiencing day time urinary frequency and 47% with nocturnal frequency [3]. Other common symptoms of fibroids include urgency (60%), stress incontinence (25%), and impaired urine flow (20–25%) [2]. Furthermore, fibroids can also affect fertility by altering the physiology of the endometrium and distorting the uterine cavity [4].

A first-line investigation for diagnosing fibroids is ultrasound, however, magnetic resonance imaging (MRI) often provides more detailed anatomical information with high resolution of images and tissue contrast [5, 6]. An additional benefit is that its performance is not impaired by shadowing and calcification, unlike ultrasound [7]. Soft tissue pelvic pathologies like fibroids are investigated with various modalities. Ultrasound is often first-line, but MRI is preferred as it provides detailed characterisation [8–11]. Fibroids are easily identified on MRI as well-defined masses of lower signal intensity on T2-weighted sequences. With sensitivity and specificity of 99% and 86%, and the ability to identify additional fibroids (mean difference 0.51 ± 1.03; P < 0.001), MRI is also valuable in assisting preoperative planning [12].

Given the increased anatomical detail, MRI has a prominent role in the investigation of various obstetric and gynaecological pathologies [13, 14]. Two-dimensional (2D) MRI can provide an accurate representation of fibroids and their anatomical relationships with other pelvic organs, however, they offer limited articulation [15]. The rendering of three-dimensional (3D) models from MRI imaging may overcome these barriers and provide better fibroid mapping through improving spatial localisation and orientation of pelvic structures.

3D imaging and modelling are increasingly utilised in various specialities, including its use in surgical planning and education [16–20]. However, while pelvic 3D modelling has been reported [14, 21], most data come from studies on computerised tomography (CT) and brain MRI images [22]. To facilitate our understanding of fibroids and LUTS, 3D imaging techniques may be employed to overcome limitations of cross-sectional imaging and possibly become part of imaging protocols in future. In the study of fibroids and LUTS, information obtained from imaging may assist in the evaluation of associations between fibroids location and size and LUTS. Stereotactic imaging may also support planning for surgical management, facilitate monitoring in conservative treatments and provide an additional modality for clinical anatomy teaching.

In this study, we aimed to assess the feasibility of rendering 3D pelvic models using cross-sectional MRI scans and to evaluate pelvic anatomy with a focus on fibroids and the lower urinary tract. Our study objective was also to compare the role and ability of two different image processing applications in the three-dimensional volume rendering based on cross-sectional MR images.

## Materials and methods

Cross-sectional images of MRI scans were obtained and analysed. These images were from 3 women with fibroids, between 36 and 58 years old, who presented with LUTS and underwent pelvic MRI between June 2016 and April 2018. Patient demographics, MRI reports and clinical information were obtained through NHS Electronic Patient Records. Demographics, symptoms and examination findings were reviewed. This study was registered as an audit (service evaluation) (ID1920WC001) at our Trust​ Audit Department.

T1- and T2-weighted images were obtained from a GE Optima 450W MRI with 1.5 Tesla magnet. Each dataset included at least one series of images from each plane (axial, coronal and sagittal). The sequence of each plane included a minimum of 20 slices, with slice thickness between 3-4 mm, slice gap between 0.3–0.4 mm and field of view between 180–320 mm. Time of acquisition was between 1–4 ms. MRI sequences were anonymised and stored in the Digital Imaging and Communication in Medicine (DICOM) format. 3D Slicer v4.8.10 and OsiriX Lite, freely available DICOM viewers of Windows and Macintosh operating systems, were used to visualise and process the sequences.

## Model rendering

Image sequences of each patient were imported into 3D Slicer and OsiriX. Pelvic structures were identified in each image sequence before processing. The following criteria were applied to delineate the pelvic organs of interest: uterus delineation includes fundus, corpus and cervix but not fallopian tubes or ovaries; bladder delineation includes the base, apex, cavity and urethral sphincter but not urethra; only the largest fibroid would be modelled in those with more than one.

3D volume rendering was performed through both 3D Slicer and OsiriX. Each structure was rendered to provide an accurate representation of the shape and size of the organs. All measurements were recorded to 0.1 mm and 1 mm^3^ precision and entered into a Microsoft Excel (2010) database for analysis. 3D modelling was achieved by segmenting the three structures across each slice where visible. For each axis of images, three labels were created to represent the uterus, urinary bladder and fibroid. The labels from each respective axis were then combined to create 3D polyhedral structures of overlapping surfaces, producing a final volume of axial, coronal and sagittal sequences. The individual rendered model of the three axes were then combined to produce a final 3D model.

## Measurements and analysis

Volumetric and dimensional measurements of the final composite models were measured within their respective applications. Shortest and longest distances were measured between superio-posterior surface of the bladder and inferio-anterior fibroid surface. Measurements of all organs were obtained from individual axes of the 3D models and used to calculate mean volumes and dimensions (height, width and depth).

## Results

All three patients presented with LUTS, two of whom had mixed urinary incontinence (MUI) and one patient had stress urinary incontinence (SUI) associated with frequency. Two patients had a sensation of incomplete bladder emptying. All three radiological reports of MRI confirmed one or more fibroids in the uterus.

**Fig. 1:**
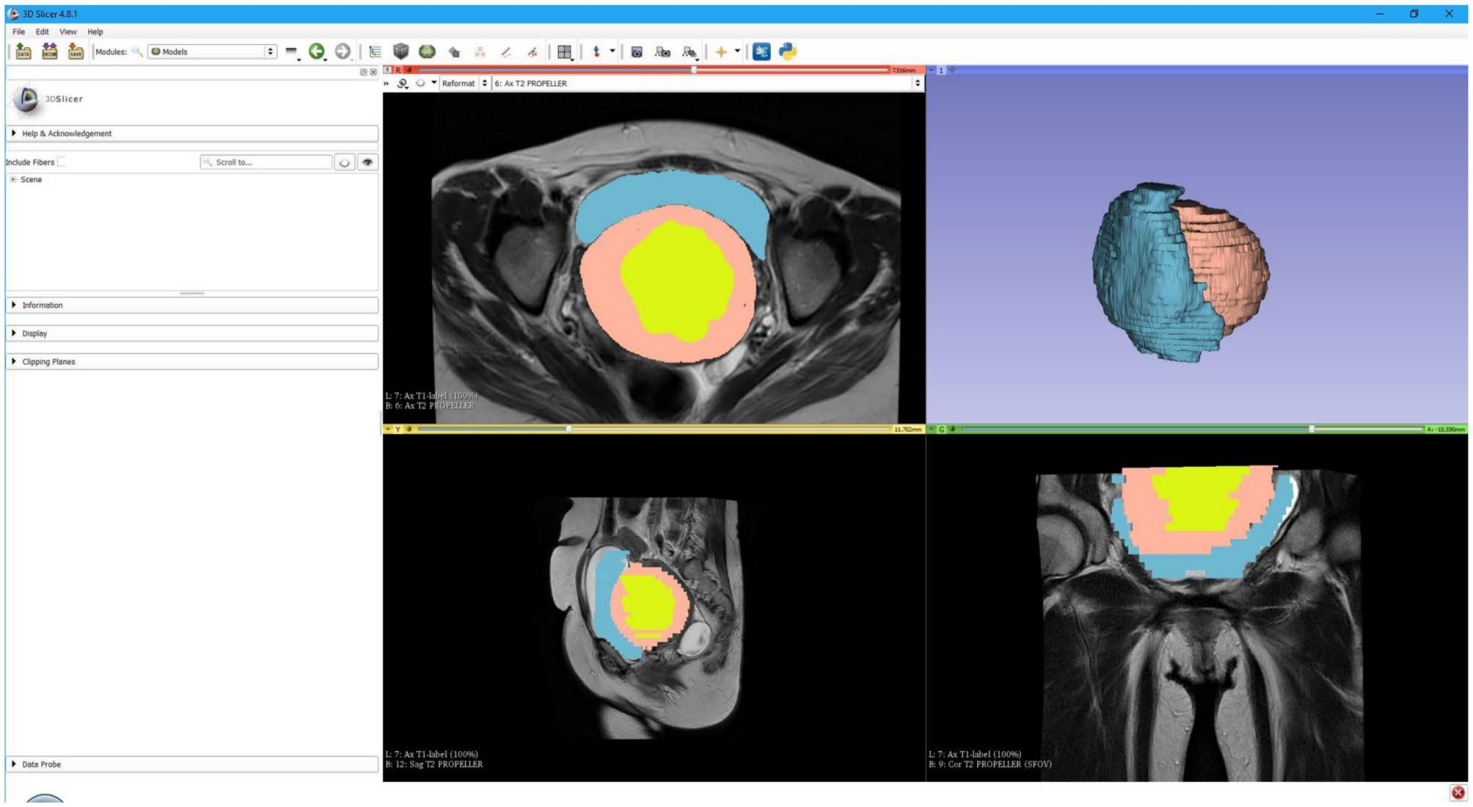
3D Slicer segmentation – Model of the bladder (blue), uterus (pink) and fibroid (lime green) displayed on the right upper corner

**Fig. 2:**
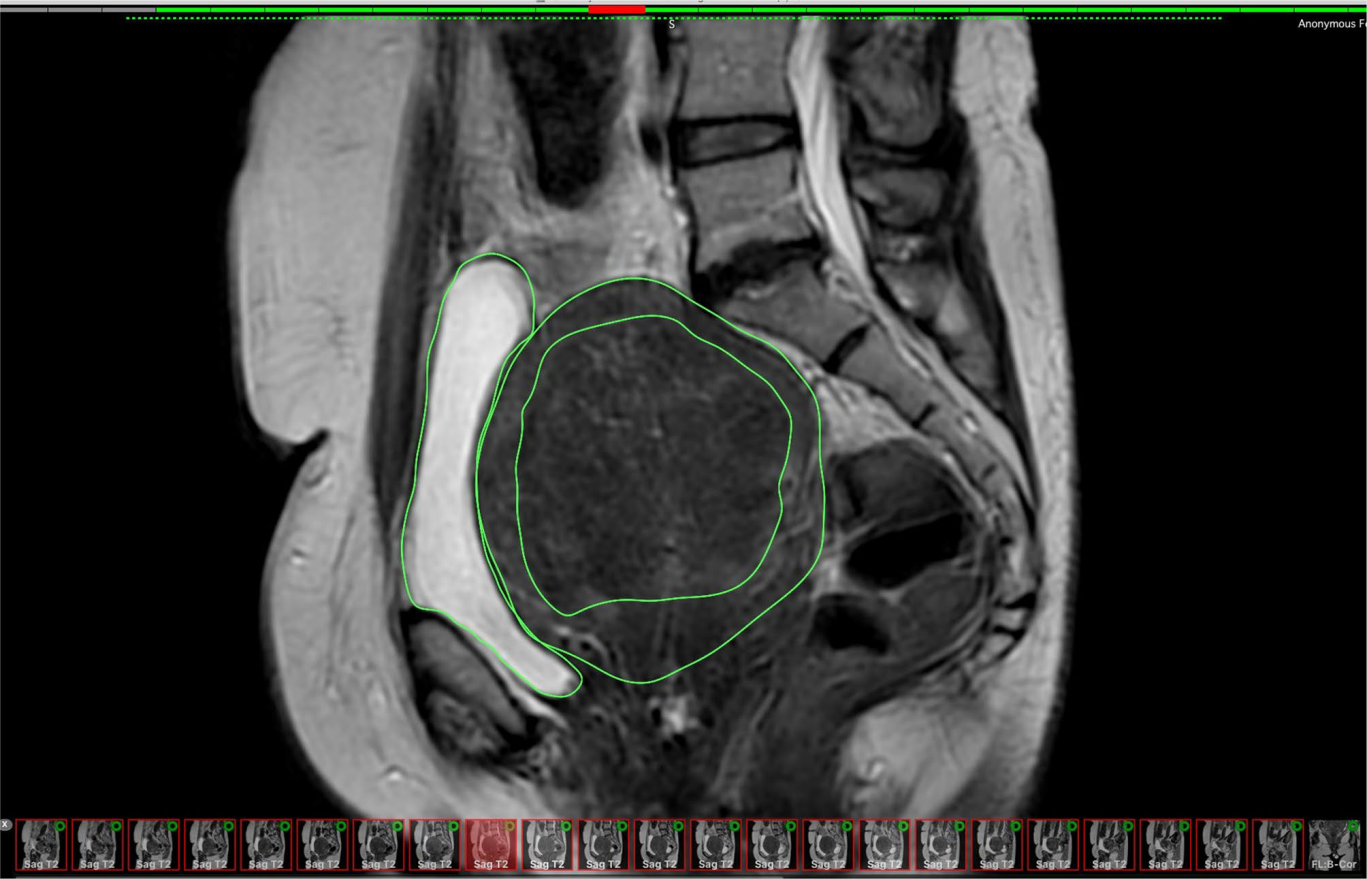
OsiriX segmentation – Superimpose ROI of uterus, bladder and fibroid

**Fig. 3:**
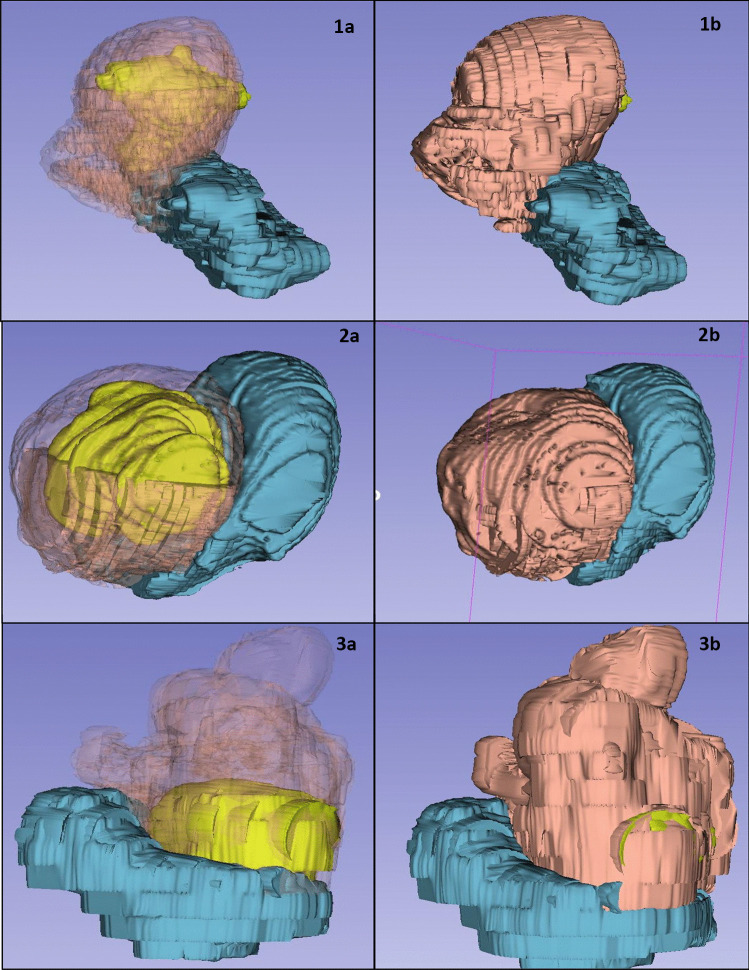
3D Slicer final models – Model of three patients with uterine fibroids bladder (blue), uterus (pink) and fibroid (lime)

**Fig. 4:**
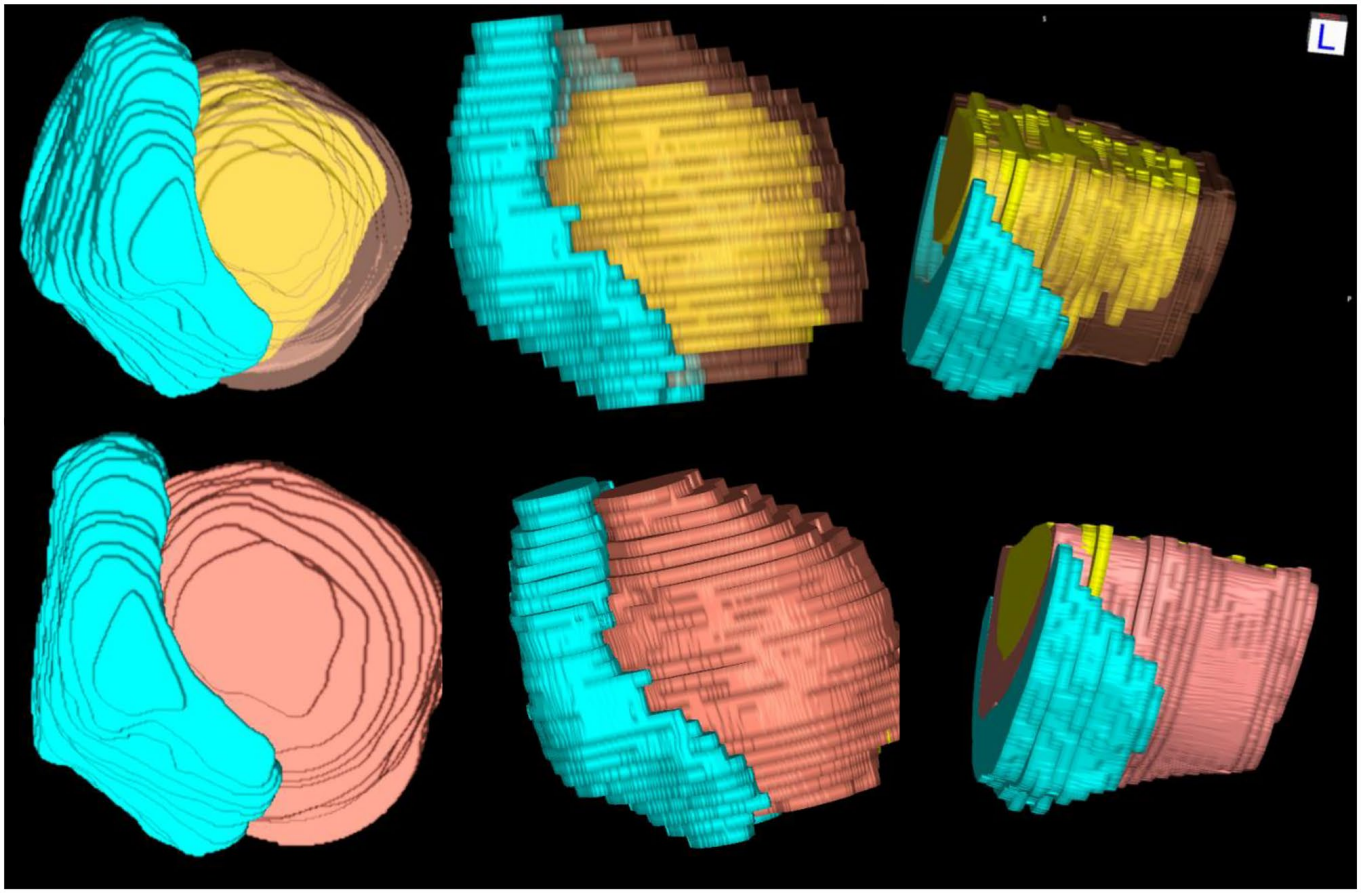
OsiriX final models – 3D models of pelvic organs of a patient (1968)- Sagittal (left), axial (centre) and coronal (right)

Organs within the models, shown in Fig. 1, were colour coded to differentiate between tissues; bladder (blue), uterus (pink) and fibroid (lime). Segmentation of the same structures via OsiriX is shown in Fig. 2. Both applications can display the 3D models of each axis separately, 3D Slicer is shown in Fig. 3 and OsiriX is shown in Fig. 4. Conversely, in 3D Slicer, the model can be viewed as a superimposed composite structure of nine structures: models of the three organs created in all three planes of images.

All models generated can be translated, rotated and scaled within the 3D display Model module within 3D Slicer and 3D Viewer function of OsiriX. The final models can also be superimposed onto the original MRI slices and allow interchange of visualisation between 2 and 3D anatomy.

## Measurements of pelvic structures

Each rendered structure of interest was measured in their respective processing applications. The mean OsiriX volume of the bladder, uterus and fibroid were 100,100 mm^3^, 192,000 mm^3^ and 73,000 mm^3^ respectively. The mean 3D Slicer volumes were 128,000 mm^3^, 215,000 mm^3^ and 63,000 mm^3^. The Spearman Coefficient of the dimensional measurements between the two applications ranged between 0.817 and 0.983. Spearman Coefficient for volumetric calculation is 0.933. Correlation was statistically significant (P-Value < 0.05). Dimensional and volumetric measurements of pelvic organs in all three patients are shown in Table 1. The measurements of the distance between the dominant fibroid and bladder surface were measured and has a Spearman Coefficients of 0.879 and 0.967 (Tables 2 and 3).

**Table 1:** Patient demographics, clinical presentation and radiological findings

Patient	Demographics	Clinical presentation	Clinical diagnosis	MRI report
1	58 years Gravida not specified	Stress incontinence, frequency, nocturia, incomplete emptying	Grade 2 cystocele or paraurethral cyst	Fibroid uterus, and nabothian cysts at cervix but no cystocele or paraurethral cyst
2	50 years Para 1, Abortus 2	Stress incontinence, frequency, nocturia, urge incontinence, incomplete emptying, urgency	Grade 1 cystocele, endometrial or fibroid polyp	7.5 cm central fundal fibroid 15 × 9 cm enlarged fibroid uterus
3	47 years Para 2, spontaneous vaginal deliveries	Stress incontinence, frequency, nocturia, urge incontinence	Detrusor overactivity	Anteverted uterus 7.3 × 6.9 × 6.3 cm with multiple fibroids Dominant fibroid in anterior intramural/subserous position, 4.5 cm transverse diameter Next largest fibroid adjacent to it, 4 cm in longest diameter

**Table 2:** Statistical analysis of fibroid measurements

Structure	Mean Height (mm)			Mean Width (mm)			Mean Depth (mm)			Mean Volume (mm^3^)		
	3D Slicer	OsiriX	Difference	3D Slicer	OsiriX	Difference	3D Slicer	OsiriX	Difference	3D Slicer	OsiriX	Difference
Patient 1												
Bladder	40.90	49.50	-8.60	84.35	85.20	-0.85	48.20	28.00	20.20	66717.00	44617.50	22099.50
Uterus	60.05	47.60	12.45	63.85	62.00	1.85	39.45	45.80	-6.35	57400.00	59343.50	-1943.50
Fibroid	25.05	22.90	2.15	41.45	25.30	16.15	25.00	21.80	3.20	6584.67	5934.50	650.17
Patient 2												
Bladder	101.05	76.50	24.55	106.50	105.50	1.00	33.20	47.80	-14.60	202250.67	140419.67	61831.00
Uterus	80.45	77.55	2.90	102.60	95.15	7.45	95.50	86.70	8.80	339583.67	345461.33	-5877.67
Fibroid	56.90	69.80	-12.90	64.35	81.75	-17.40	79.05	76.60	2.45	132208.67	163216.67	-31008.00
Patient 3												
Bladder	54.45	58.70	-4.25	88.00	91.70	-3.70	70.65	77.70	-7.05	115200.33	117901.00	-2700.67
Uterus	90.15	75.90	14.25	80.20	74.20	6.00	84.00	82.20	1.80	248275.33	170461.00	77814.33
Fibroid	43.70	43.60	0.10	45.35	49.60	-4.25	50.60	51.10	-0.50	51577.33	49789.00	1788.33
Spearman Coefficient	0.817			0.983			0.917			0.933		
P-Value	0.007			< 0.001			0.001			< 0.001

**Table 3:** Statistical analysis of spatial relation between fibroid and bladder surface

Patient	Image Axis	Mean distance between bladder and proximal fibroid contact surface (mm)			Mean distance between bladder and distal fibroid contact surface (mm)		
		3D Slicer	OsiriX	Difference	3D Slicer	OsiriX	Difference
1	Axial	16.75	11.50	5.25	34.61	32.80	1.81
	Coronal	19.20	10.80	8.40	36.07	41.10	-5.03
	Sagittal	17.06	12.70	4.36	33.90	36.80	-2.90
2	Axial	4.20	9.50	-5.30	66.20	83.60	-17.40
	Coronal	3.40	5.20	-1.80	64.40	81.70	-17.30
	Sagittal	3.50	4.30	-0.80	56.50	70.40	-13.90
3	Axial	0.20	0.80	-0.60	47.80	44.00	3.80
	Coronal	0.40	0.50	-0.10	46.80	41.50	5.30
	Sagittal	0.40	0.30	0.10	48.20	43.50	4.70
Spearman Coefficient		0.879			0.967		
P-Value		0.002			< 0.001		

## Discussion

In this study, 3D models of pelvic structures were successfully rendered within both image processing applications OsiriX and 3D Slicer. 3D composites of bladder, uterus and fibroid can be measured, rotated and manipulated in real-time to provide information on anatomical characteristics and relationships. The models allow visualisation of volume, the position of the dominant fibroid and its relative position to the bladder. Both open-source applications were user-friendly in our opinion and include customisations to optimise segmentation, rendering and visualisation. Our study demonstrated that despite differences in the segmentation process, measurements made with the two applications correlated strongly with each other throughout all dimensional and volumetric measurements.

MRI is increasingly used in the assessment and treatment of fibroids. Recent research highlights the applicability of fibroid anatomy in predicting symptoms and treatment responsiveness over patient characteristics [23–25]. Whilst volume of the uterus and fibroids were thought to impact LUTS, Koch et al*.* found no significant correlation but noted an association between anterior/fundal fibroids and OAB, suggesting that position also affect LUTS development. The lack of literature focussing on spatial relationships of pelvic organs likely contributes to the discrepancies in the understanding of LUTS, as the majority of available literature remains limited to cross-sectional imaging and highlights the need to explore the full potential of 3D MRI.

With advances in MRI, unprecedented levels of anatomical detail can be reached with many applications. The creation of 3D models through segmenting and merging images can offer an improved signal-to-noise ratio over planar imaging and help overcome artefact interference [26]. 3D reconstruction of fibroid position for surgical planning has been reported [26]. However, the study by Lee et al., was focused on localisation on a 3D gird principle. To our knowledge there is no literature evaluating specifically 3D fibroid’s position in relation to LUTS symptoms.

To create 3D models, various image processing applications have been developed and may be used. Virzì et al. highlighted technical properties such as automatisation, usability, and segmentation time to be variable between tools. Apart from 3D Slicer and OsiriX used in this study, other tools including Amira, Anatomist, FSL, ITK-SNAP, Myrian Studio and Seg 3D are available, with the majority being freely accessible [22]. Based on the usability in managing and manipulation of models, 3D Slicer, OsiriX, Myrian Studio and ITK-SNAP were all considered the best for 3D visualisation [22]. The visualisations achieved by 3D Slicer and OsiriX were user-friendly and intuitive with clear and succinct interfaces, whereas tools like Anatomist and Image J scored poorly. We confirmed in this study that both 3DSlicer and OsiriX are excellent tools for fibroid 3D reconstruction and correlation to LUTS symptoms.

In fibroid management, 3D models can better localise and characterise thus facilitating our diagnostic ability and visualisation of response to treatment [27]. 3D models, using both CT and MRI, have also value in surgical interventions such as myomectomy and nephrectomy operations as the use of these modalities could facilitate surgical planning [17, 28]. However, as the uses of 3D models in pelvic pathology remain limited, the results from this study supports their utility. The rendered models can be used to accurately demonstrate anatomical relationships between pelvic organs as well as provide a means to make measurements where necessary. This additional information may be further utilised in the clinical, perioperative and educational aspects of LUTS and fibroids. These benefits have been explored, and shown to be advantageous, in other surgical specialities.
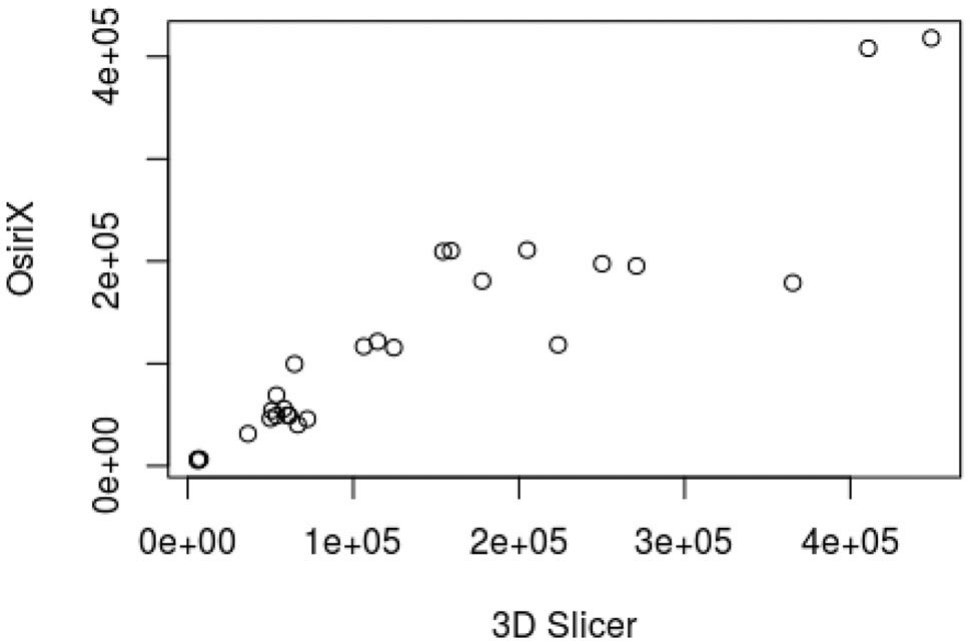



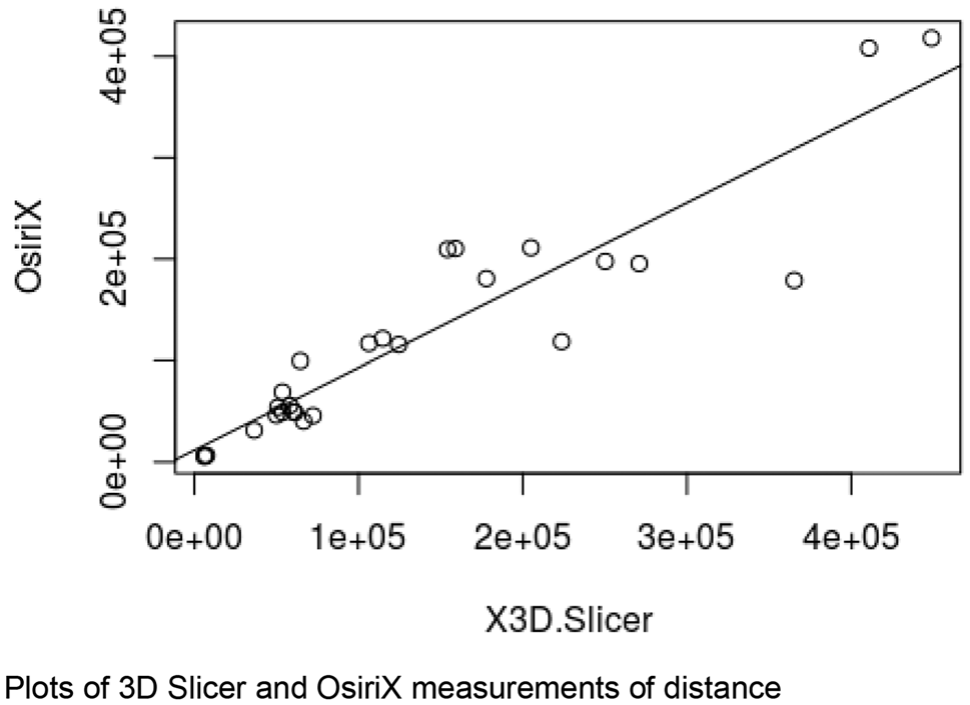



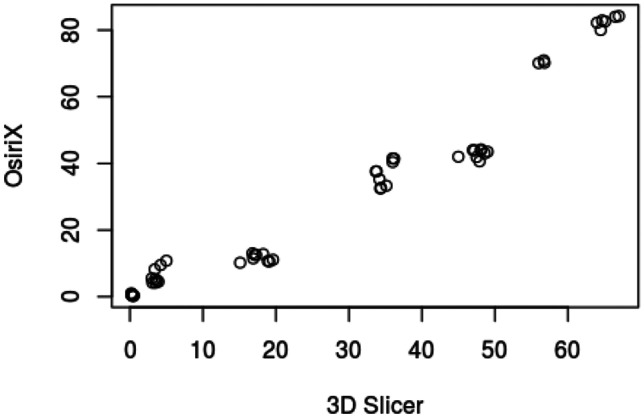



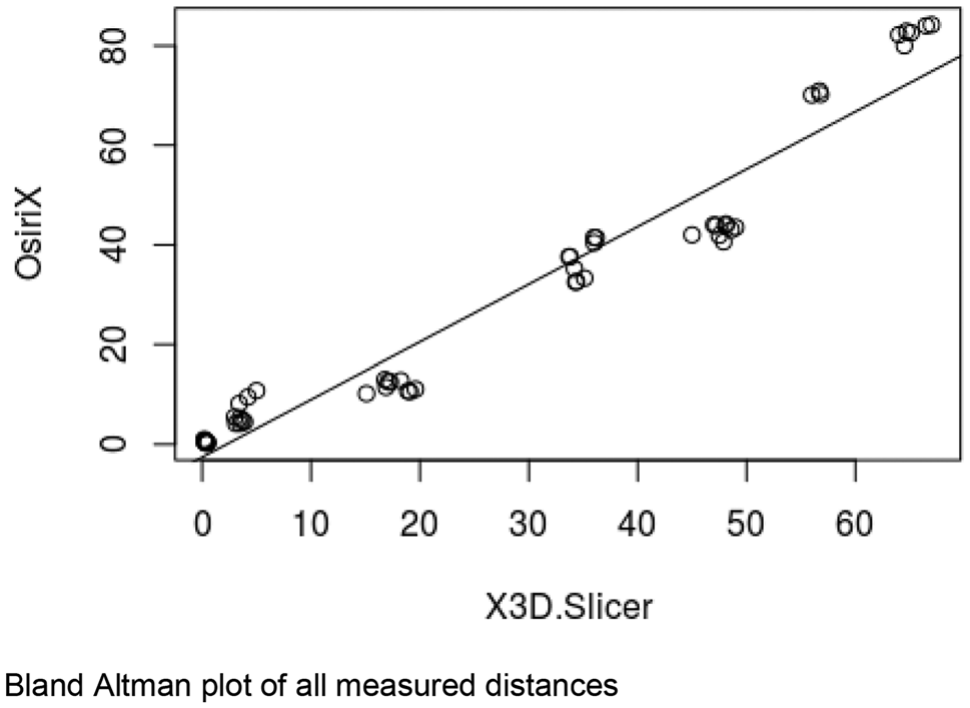



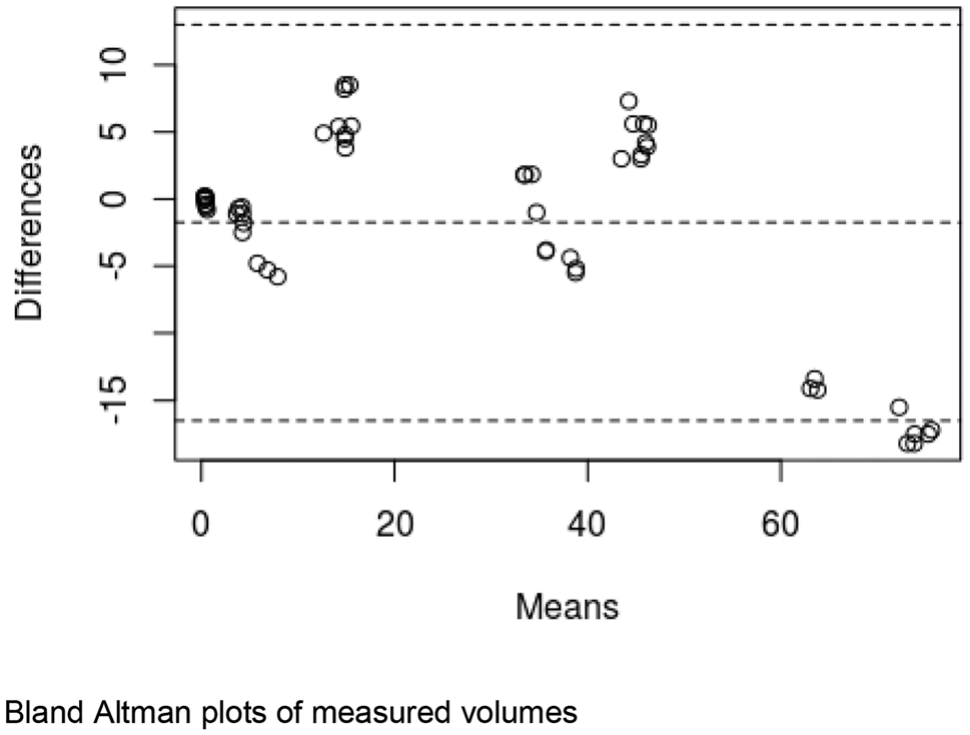



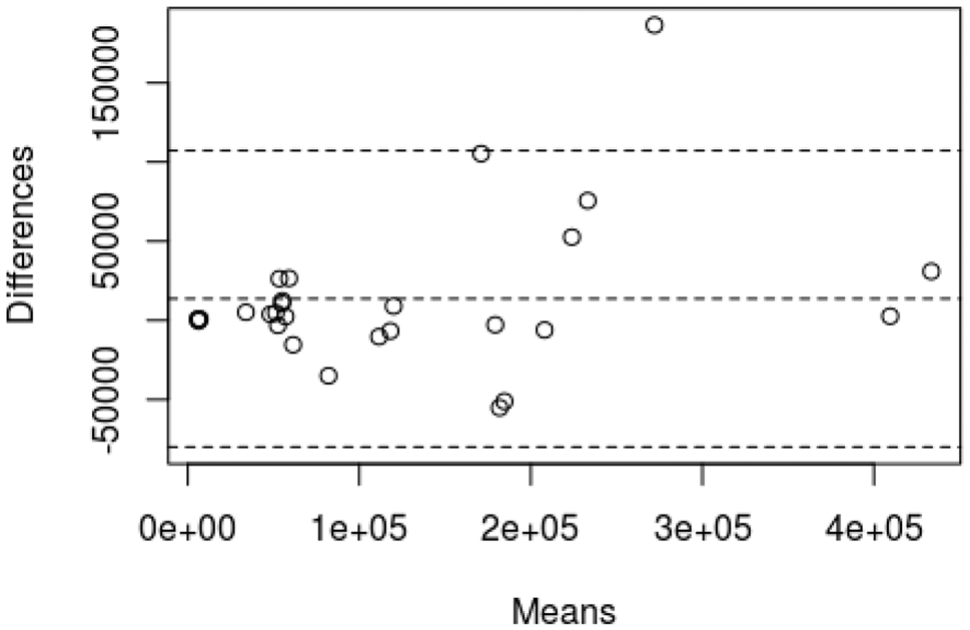


## Strengths

To our knowledge this is the first study investigating the feasibility of 3D anatomical models of fibroids in relation to the lower urinary tract using two different image processing applications and comparing the measurements obtained. We followed a standardised approach that our research group has developed and applied in anatomical and image processing studies of the pelvic floor [14, 21].

## Limitations

This study may carry inherent limitations due to its methodology as a feasibility-only study. Hence, generalisability of our findings remains to be confirmed in larger-scale studies. The MRI sequences obtained here may not be representative of sequences used elsewhere, which may affect the applicability of our observations in different MR imaging specifications.

## Conclusion

This study confirms the feasibility of creating 3D models of pelvic organs from routinely obtained MRI scans. These models provide detailed and previously unobtainable anatomical measurements of bladder, uterus and fibroids. Rendered 3D models provide a better understanding of how fibroids relate to specific pelvic organs and anatomical structures and may prove an additional diagnostic modality in women with fibroids and LUTS. It may offer relevant clinical information which could assist in improving the management of these women. Both image processing applications (OsiriX and 3D Slicer) delivered good quality volume rendering of sequential 2D images with good correlation in measurements in this study. However, more research is needed to optimise imaging protocols and possibly to maximise both image processing efficiency and model accuracy.

## Data Availability

Data available upon reasonable request.

## Declarations

### Presentations

Poster presentations at the International Urogynecological Association 2019 Annual Meeting and the Royal College of Surgeons of Edinburgh 2020 International Undergraduate Conference.
